# Supplementary material for: Central Asian Rodents as Model Animals for Leishmania major and Leishmania donovani Research
Source: Microorganisms. 2020 Sep 20;8(9):1440. doi: 10.3390/microorganisms8091440 (PMC7563294; doi:10.3390/microorganisms8091440)
Supplement: Supplementary file 1 [file microorganisms-08-01440-s001.pdf]

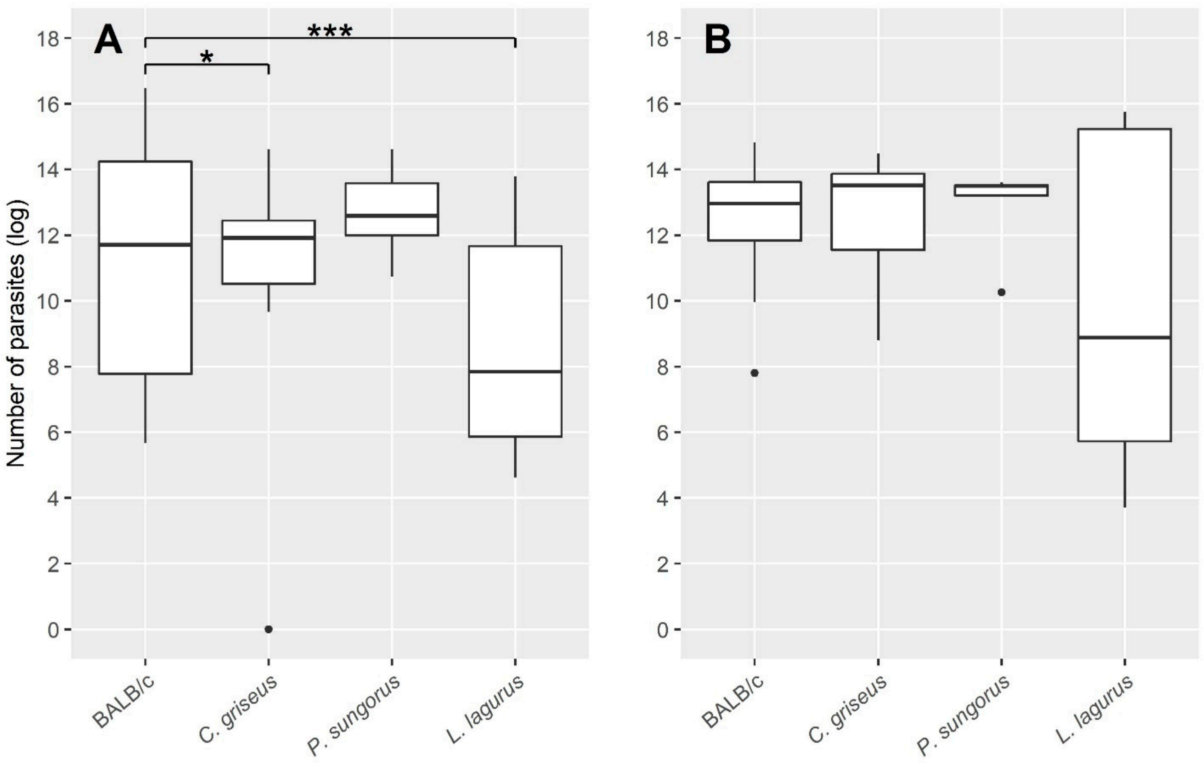

Figure S1. Numbers of *L. major* parasites in inoculated ears evaluated by Q PCR (**A**) and flow cytometry (**B**). Data are presented as natural logarithms and are shown in box plot graphs where boxes display 25th-75th percentiles and horizontal line represent the median value; vertical lines represent minimum to maximum values and full circles outliers. Asterisks indicate significant difference between BALB/c mice set as reference and other species; * = $P < 0.05$; *** = $P < 0.001$.

Table S1. Weight gains in four rodent species inoculated with *L. major*.
CX, rodents inoculated with CDP, SX, rodents inoculated with SDP. *, animals dying before the end of the experiment. *P*, significance of the difference between CDP and SDP groups.

Rodent species	Week p.i.	**C1**	**C2**	**C3**	**C4**	**C5**	**C6**	**S1**	**S2**	**S3**	**S4**	**S5**	**S6**				
**BALB/c mice**	1	18	19	21	21	23	23	21	22	22	21	20	20				
***P* = 0.82**	2	18	19	21	22	22	24	22	22	21	22	20	21				
	3	18	20	22	23	24	24	22	23	22	21	20	21				
	4	18	21	21	23	25	23	23	23	23	21	21	22				
	5	20	22	23	22	27	24	22	23	21	22	22	23				
	6	20	22	23	23	25	23	23	24	22	23	23	23				
	7	21	24	24	23	26	24	23	25	22	24	23	23				
	8	21	23	23	23	26	24	23	24	22	21	20	21				
	9	21	21	19	23	24	23	24	24	23	22	23	23				
	10	22	22	22	23	24	24	24	24	24	23	22	23				
	11	22	21	21	23	24	24	24	24	24	22	21	20				
C. griseus		C1	C2	C3	C4	C5	C6	C7	C8*	S1	S2	S3	S4	S5	S6	S7*	S8*
***P* = 0.69**	1	26	32	27	26	30	27	28	27	26	26	20	26	25	32	33	28
	2	26	32	27	26	30	27	27	28	26	26	22	26	24	32	32	26
	3	26	32	27	27	29	28	28	27	25	26	22	25	23	31	x	26
	4	26	32	26	26	28	28	27	27	26	27	23	25	24	32	x	26
	5	28	33	27	27	28	29	28	27	26	26	26	25	23	31	x	25
	6	28	33	26	27	27	29	28	28	26	27	24	25	24	30	x	26
	7	29	34	26	27	27	29	28	x	26	27	24	24	24	31	x	25
	8	29	34	27	27	27	29	28	x	26	27	25	25	23	31	x	26
	9	30	35	28	26	25	30	29	x	27	28	25	26	24	28	x	25
	10	30	37	27	26	26	29	28	x	25	24	23	25	21	26	x	x
	11	28	35	25	27	27	27	26	x	26	25	26	26	24	29	x	x
	12	30	36	27	26	27	29	26	x	27	26	26	26	24	32	x	x
	13	31	37	27	27	26	29	26	x	27	28	25	26	24	31	x	x
	14	31	36	28	26	27	29	27	x	27	27	25	27	24	32	x	x
	15	30	36	27	27	27	29	27	x	27	26	26	26	25	32	x	x
P. sungorus		C1	C2	C3	C4	C5	C6	S1	S2	S3	S4	S5	S6*				
***P* = 0.22**	1	35	32	38	33	29	32	30	32	30	45	26	24				
	2	37	30	40	33	27	31	32	30	31	46	25	25				
	3	37	32	43	30	28	30	34	33	34	45	26	26				
	4	39	34	42	31	24	30	35	32	33	48	25	29				
	5	41	37	44	30	21	29	37	34	32	48	23	x				

	6	43	36	46	29	22	28	39	36	31	48	25	x				
	7	44	37	44	29	22	28	41	37	33	48	26	x				
	8	45	35	46	28	20	27	43	38	32	49	25	x				
	9	47	37	45	28	22	27	42	39	33	49	27	x				
	10	50	37	45	27	21	27	41	43	33	48	28	x				
	11	x	x	x	27	21	27	x	x	x	50	28	x				
L. lagurus		C1	C2	C3	C4	C5	C6	C7	C8	S1	S2	S3	S4	S5	S6	S7	S8
***P* = 0.66**	1	32	26	34	30	24	36	27	31	30	27	32	30	38	31	30	32
	2	33	27	33	31	25	37	27	30	32	25	32	31	38	31	29	33
	3	31	28	34	28	25	36	28	33	32	26	34	33	38	35	31	33
	4	30	29	33	26	26	39	29	33	31	27	33	35	39	34	33	35
	5	29	29	32	26	25	40	28	32	33	28	31	34	40	34	33	34
	6	29	28	35	26	26	40	27	34	35	28	33	33	41	33	35	34
	7	30	29	35	25	25	42	29	34	35	30	35	32	40	33	35	35
	8	30	27	34	25	25	41	29	35	34	33	37	31	39	32	36	34
	9	31	30	34	26	25	41	30	35	32	36	37	31	40	33	35	34
	10	30	32	34	26	26	40	31	36	33	39	36	32	40	33	36	34
	11	31	33	35	26	27	41	30	37	33	37	36	33	42	32	37	34
	12	33	35	34	37	25	43	30	38	35	35	36	34	44	32	39	33
	13	32	35	34	37	26	42	32	38	36	35	38	34	43	31	38	33
	14	33	34	33	36	26	41	31	39	38	34	39	33	44	31	39	34
	15	35	36	33	37	27	41	30	39	39	35	39	34	44	31	39	34

Table S2. Lesions diameters (mm) in animals inoculated with *L. major*.

**Rodent species**	**Week p.i.**	**C1**	**C2**	**C3**	**C4**	**C5**	**C6**	**S1**	**S2**	**S3**	**S4**	**S5**	**S6**				
**BALB/c mice**	1	0	0	0	0	0	0	0	0	0	0	0	0				
***P* = 0.97**	2	0	0	0	3.9	2.3	2.4	0	0	0	0	0	0				
	3	1	1	1	4.7	3.6	3.5	0	0	0	0	0	0				
	4	2.8	2.7	2.6	5.4	4.6	5.1	0	1	1	1	0	0				
	5	2.9	2.9	4.3	7	5.5	6	0	3.8	2.9	1	0	0				
	6	3.2	4.1	4.3	7.5	6.5	7	0	4.6	4.5	2.6	1	1				
	7	4	5	4.4	11	7.6	10	1.4	4	5.2	4.8	5.8	3				
	8	5	6.5	7.3	11	11	11	2.8	3.9	5.3	7.8	8	8				
	9	7.8	9.4	10	11	11	11	5.3	4.3	6	8	8.7	8.5				
	10	8	9.5	10	x	x	x	x	x	x	9	10	9.3				
	11	9.5	12	10.7	x	x	x	x	x	x	9.5	11.6	10				
C. griseus		C1	C2	C3	C4	C5	C6	C7	C8*	S1	S2	S3	S4	S5	S6	S7*	S8*

***P* = 0.07**	1	0	0	0	0	0	0	0	0	0	0	0	0	0	0	0	0
	2	0	0	0	0	0	0	0	0	0	0	0	0	0	0	0	0
	3	0	0	0	0	0	0	0	0	0	0	0	0	0	0	x	0
	4	1	0	0	0	0	1	0	0	0	0	0	0	0	0	x	0
	5	1.4	2.9	1.8	2.7	2.2	2.2	1	1	0	0	0	0	0	0	x	0
	6	2	3	2	3.5	3	3.7	2	3.8	0	0	0	0	0	0	x	0
	7	2.4	3.7	2.7	5.4	3.6	4	2	x	1.2	0	0	1	0	1.8	x	0
	8	4	6.2	5.3	7	5	6	4.6	x	3	0	0	2.5	0	3	x	0
	9	4.5	5.2	4.6	10	4.3	5.5	5.6	x	2.6	0	0	2.3	1	2.5	x	1.9
	10	4.8	4.7	5	10	5	5	5.6	x	2	0	0	2	2.4	2.1	x	x
	11	6.5	5.5	5.3	10	10	7	6.5	x	2	1	0	2.5	6.5	3	x	x
	12	7	6	5.3	10	10	8.7	6.4	x	1.8	1.5	1	2.3	2.6	4.3	x	x
	13	8.5	6.7	5.7	9	8	9	8.3	x	1	3,7	1	2	1.8	4.9	x	x
	14	10	8.5	3	8.5	10	10	10	x	0	4	1	2	1	5.5	x	x
	15	11	8.7	2.3	9	11	10.3	11	x	0	5.5	0	2.1	0	6.7	x	x
P. sungorus		C1	C2	C3	C4	C5	C6	S1	S2	S3	S4	S5	S6*				
***P* = 0.68**	1	0	0	0	0	0	0	0	0	0	0	0	0				
	2	0	0	0	0	0	0	0	0	0	0	0	0				
	3	0	0	0	0	2.5	1	0	0	0	1	2	3				
	4	1	1	1	0	4	1.6	0	0	0	4	3.7	5.2				
	5	4.7	5.1	4	3	4.4	4.6	4.5	0	4.87	4	6.5	x				
	6	4.2	5.9	5.4	4.6	5	4.6	5.2	3.6	5.8	7.6	7.8	x				
	7	4	4.2	5.7	4.5	5.3	4.1	4.8	5	3.7	5.5	6.6	x				
	8	5.3	9	5.8	4.8	6	4.7	9	5	8	6	6.4	x				
	9	4.5	11	6	5.5	7	7	11	7	8	7.4	5	x				
	10	5.6	8.7	7	6.4	7.3	6.8	8	7.3	8.8	7	5.9	x				
	11	x	x	x	7	7.8	6.5	x	x	x	6.8	6.3	x				
L. lagurus		C1	C2	C3	C4	C5	C6	C7	C8	S1	S2	S3	S4	S5	S6	S7	S8
***P* = 0.69**	1	0	0	0	0	0	0	0	0	0	0	0	0	0	0	0	0
	2	0	0	0	0	0	0	0	0	0	0	0	0	0	0	0	0
	3	0	0	0	5.3	5.6	4	3.7	4.5	0	0	0	4.6	3.8	4.1	4.3	5.6
	4	1	1	1	3.8	4.7	5.1	5.8	4.8	0	0	0	3.3	4	4	7	3.8
	5	3.16	4.3	4.5	4.1	3.3	4.1	4.6	2.3	3.3	3.2	0	3.2	4.1	5.5	6.2	4.3
	6	3	5	4.6	2.1	3.2	4.1	3.8	5.2	4.4	3.7	0	2.5	2.2	2	5.6	2.4
	7	4.2	4.2	5.7	3.5	1.6	4.7	2.7	2	4.1	3.3	3	2.2	3.6	4.5	6	2.8
	8	4.2	4.4	4.6	1.8	1.6	2.2	2.6	1.5	3.8	4	3.3	1.3	3.4	4.4	4.4	2.8
	9	4.5	5	4.2	1.5	1.5	2	1.9	1	6	6	0	0	1.8	4	4.5	2.6
	10	3.5	4.5	3.5	1.2	1.2	2	1.5	1.2	5.3	5	0	0	2	3.9	4.3	2.3
	11	3.4	4.3	3	1	1	1.2	1	1	5	4.3	0	0	1.5	3.8	4	2
	12	3.1	4.1	2.7	1	1	1	1	1	4.6	4	0	0	1	2.3	2.5	1
	13	3	4	2.1	1	0	0	0	0	4	3.5	0	0	0	2	2	1
	14	2.8	3.9	1.4	0	0	0	0	0	3	3.4	0	0	0	0	0	0
	15	0	1.5	0	0	0	0	0	0	4	2	0	0	0	0	0	0

CX, rodents inoculated with CDP, SX, rodents inoculated with SDP. *, animals dying before the end of the experiment. P, significance of the difference between CDP and SDP groups.

Table S3. *Leishmania* loads and distribution determined by qPCR in animals inoculated with *L. major*.

Rodent species	Tissues and organs	CDP						SDP							Tissues and organs	CDP								SDP							
BALB/c mice		**C1**	**C2**	**C3**	**C4**	**C5***	**C6**	**S1**	**S2**	**S3**	**S4**	**S5**	**S6**	*C. griseus*		**C1**	**C2**	**C3**	**C4**	**C5**	**C6**	**C7**	**C8***	**S1**	**S2**	**S3**	**S4**	**S5**	**S6**	**S7***	**S8***
	**B**	0	0	0	0	0	0	0	0	0	0	0	0		**B**	0	0	0	0	0	0	0	0	0	0	0	0	0	0	0	0
	**IE**	++	++	+	+++	++++	++	++	+	++	+++	+++	+++		**IE**	+++	+++	+++	++	0	++	++	0	+++	++	+++	++	+++	+++	+	++
	**CE**	0	0	+	++	++	0	0	0	0	++	++	++		**CE**	++	++	0	++	0	++	+++	NA	++	++	+	++	+	0	0	0
	**LN-IE**	+	+	+	++	+++	++	+	+	+	++	++	++		**LN-IE**	+++	+++	+++	NA	NA	NA	NA	0	++	+++	+++	0	NA	NA	NA	0
	**LN-CE**	0	0	+	0	++	0	0	+	0	0	0	+		**LN-CE**	++	++	++	NA	NA	0	NA	0	0	++	++	0	NA	0	NA	NA
	**FP**	0	0	0	0	0	0	0	0	0	0	0	0		**FP**	0	++	0	0	0	0	0	0	0	++	+	0	0	++	0	0
	**HP**	0	0	0	0	0	0	0	0	0	0	0	0		**HP**	++	+	0	0	0	0	0	0	0	+	0	0	0	+	0	0
	**T**	0	0	0	0	0	0	0	0	0	0	0	0		**T**	0	+	+	0	0	0	0	0	+	+	+	0	0	+	0	0
	**L**	0	0	0	0	0	0	0	0	0	0	0	0		**L**	0	++	0	0	0	0	0	0	0	0	+	0	0	++	0	0
	**S**	0	0	0	0	0	0	0	0	0	0	0	0		**S**	++	++	++	0	0	0	0	0	++	0	+	0	0	++	0	0
P. sungorus		**C1**	**C2**	**C3**	**C4**	**C5**	**C6**	**S1**	**S2**	**S3**	**S4**	**S5**	**S6***	*L. lagurus*		**C1**	**C2**	**C3**	**C4**	**C5**	**C6**	**C7**	**C8**	**S1**	**S2**	**S3**	**S4**	**S5**	**S6**	**C7**	**C8**
	**B**	0	0	+	++	++	0	0	0	++	+	0	NA		**B**	0	0	0	0	0	0	0	0	0	0	0	0	0	0	0	0
	**IE**	+++	++	+++	+++	++	+++	+++	+++	+++	+++	+++	NA		**IE**	++	++	+++	+	+	+	+	+	+++	+++	+++	++	++	+	+	++
	**CE**	++	++	0	++	++	++	+	++	++	++	++	NA		**CE**	+	++	0	+	+	+	+	+	++	++	++	+	+	+	+	+
	**LN-IE**	++	+++	+++	0	+++	+++	+++	+++	+++	+++	+++	NA		**LN-IE**	0	+++	+	0	0	0	+	0	0	+	+	0	0	0	0	0
	**LN-CE**	0	++	++	++	+	+	++	++	++	0	0	NA		**LN-CE**	+	0	0	0	0	0	0	0	0	0	0	0	0	0	0	0
	**FP**	0	++	0	0	++	++	+	0	++	+++	+++	NA		**FP**	+	++	+++	0	0	0	0	+	++	++	++	0	0	0	0	0
	**HP**	0	+	0	++	++	++	0	++	+	++	++	NA		**HP**	++	0	+++	0	+	0	0	0	++	0	+	0	0	0	0	0
	**T**	+	+	+	++	++	++	+	0	+	++	++	NA		**T**	0	0	0	0	0	0	0	0	0	+	0	0	0	0	0	0
	**L**	0	0	0	+	0	+	+	0	++	0	0	NA		**L**	0	0	0	0	0	0	0	0	0	0	0	0	0	0	0	0
	**S**	++	0	++	++	++	++	+	++	++	0	0	NA		**S**	+	0	0	0	0	0	0	0	0	0	0	0	0	0	0	0

CDP - Rodents inoculated with culture-derived parasites, SDP - rodents inoculated with sand fly -derived parasites. *, animals dying before the end of the experiment. NA, not analyzed. IE, inoculated ear; CE, contralateral ear; LN-IE, draining lymph nodes of the inoculated ear; LN-CE, draining lymph nodes of the contralateral ear; FP, forepaws; HP, hindpaws; T, tail; L, liver; S, spleen; B, blood; +, $< 10^3$ parasites; ++, $\geq 10^3$ and $< 10^5$ parasites; +++, $\geq 10^5$ and $< 10^7$ parasites; ++++, $\geq 10^7$ parasites.

Table S4: Weight gains in four rodent species inoculated with *L.donovani.*

**Rodent species**	**Week p.i.**	**C1**	**C2**	**C3**	**C4**	**C5**	**C6**	**C7**	**C8**	**S1**	**S2**	**S3**	**S4**	**S5**	**S6**	**S7**	**S8**
**BALB/c mice**	1	22	22	22	26	25	25	25	26	22	22	22	25	25	25	25	25
***P* = 0.50**	2	22	23	22	26	26	26	26	26	22	23	22	26	26	26	26	26
	3	22	23	22	27	26	29	26	27	22	23	22	26	26	26	26	26
	4	23	23	23	27	26	27	26	27	23	23	23	25	26	26	27	25
	5	24	24	24	27	27	29	27	27	24	24	24	25	27	26	27	25
	6	24	24	25	27	28	30	28	27	24	24	25	26	27	27	27	26
	7	25	25	25	28	28	30	28	28	25	25	25	26	28	28	27	26
	8	25	25	26	28	29	31	28	28	25	25	26	27	28	28	28	27
	9	25	24	26	28	29	31	28	28	25	24	26	27	28	29	28	27
	10	24	26	25	28	29	31	27	28	24	26	25	27	28	29	28	27
	11	25	26	26	28	29	31	27	28	25	26	26	27	28	29	28	27
	12	25	26	26	28	29	31	28	28	25	26	26	27	28	29	28	27
	13	25	25	26	29	29	31	28	29	25	25	26	27	29	30	29	27
	14	25	26	27	29	29	31	28	29	25	26	27	27	29	30	29	27
	15	25	26	27	29	29	31	28	29	25	26	27	28	29	29	29	28
	16	x	x	x	29	29	31	27	29	x	x	x	27	27	29	29	27
	17	x	x	x	29	29	31	28	29	x	x	x	27	28	29	29	27
	18	x	x	x	29	29	31	29	29	x	x	x	27	28	30	28	27
	19	x	x	x	29	29	31	29	29	x	x	x	28	28	30	29	28
	20	x	x	x	29	29	31	29	29	x	x	x	28	28	30	29	28
	21	x	x	x	29	29	31	29	29	x	x	x	28	28	30	29	28
	22	x	x	x	29	29	31	30	29	x	x	x	28	28	30	29	28
	23	x	x	x	29	29	31	30	29	x	x	x	28	29	30	30	28
	24	x	x	x	29	29	31	30	29	x	x	x	28	29	31	30	28
	25	x	x	x	29	x	31	30	29	x	x	x	28	29	31	30	28
	26	x	x	x	29	x	31	30	29	x	x	x	28	29	31	30	28
	27	x	x	x	29	x	31	30	29	x	x	x	28	29	31	30	28
	28	x	x	x	29	x	31	x	29	x	x	x	28	30	31	30	28
	29	x	x	x	29	x	31	x	29	x	x	x	28	30	31	30	28
	30	x	x	x	29	x	31	x	29	x	x	x	28	30	31	30	28
**Chinese hamsters**		C1	C2	C3	C4	C5	C6	C7	C8	S1	S2	S3	S4	S5	S6	S7	S8
***P* = 0.70**	1	42	35	35	34	42	37	39	34	37	43	41	37	34	39	33	37
	2	42	35	36	34	42	37	40	34	37	43	41	38	36	39	34	38
	3	43	35	36	35	42	37	41	35	37	43	42	39	36	39	34	39
	4	42	36	37	36	42	37	40	36	36	43	42	38	37	38	34	38
	5	42	36	36	37	41	37	42	37	36	43	42	38	36	39	35	38
	6	42	36	38	37	42	37	42	37	36	43	43	38	36	39	36	38
	7	41	35	35	37	42	37	42	37	35	41	42	39	37	39	37	39
	8	42	36	37	38	42	37	42	38	36	43	43	39	37	39	37	39
	9	43	37	36	38	43	37	41	38	37	44	44	39	37	39	37	39

	10	43	37	37	38	43	37	41	38	38	43	44	39	37	39	37	39
	11	44	38	38	38	43	37	41	38	38	44	43	39	37	39	37	39
	12	44	38	38	38	43	37	41	38	38	45	43	39	37	39	37	39
	13	44	38	37	38	43	37	41	38	39	44	43	39	37	39	37	39
	14	44	40	39	38	43	37	41	38	40	45	44	39	37	39	37	39
	15	45	45	42	40	45	39	42	40	42	45	45	42	39	41	37	42
	16	x	x	x	39	44	39	41	39	x	x	x	40	38	40	37	40
	17	x	x	x	39	44	39	41	39	x	x	x	40	38	40	37	40
	18	x	x	x	39	44	39	41	39	x	x	x	40	38	40	37	40
	19	x	x	x	39	44	39	41	39	x	x	x	40	37	40	38	40
	20	x	x	x	39	44	38	42	39	x	x	x	41	37	40	38	41
	21	x	x	x	39	44	38	42	39	x	x	x	41	37	40	38	41
	22	x	x	x	39	44	38	42	39	x	x	x	41	37	40	38	41
	23	x	x	x	39	45	38	42	39	x	x	x	41	37	40	38	41
	24	x	x	x	39	45	38	42	39	x	x	x	41	37	40	38	41
	25	x	x	x	40	45	38	42	40	x	x	x	41	37	40	38	41
	26	x	x	x	40	45	37	42	40	x	x	x	41	37	41	38	41
	27	x	x	x	40	45	37	42	40	x	x	x	41	37	41	39	41
	28	x	x	x	40	45	37	42	40	x	x	x	41	37	41	39	41
	29	x	x	x	40	45	37	42	40	x	x	x	41	37	41	39	41
	30	x	x	x	40	45	37	42	40	x	x	x	41	38	41	39	41
M. auratus		C1	C2	C3	C4*	C5*	C6*	S1	S2	S3	S4	S5	S6				
***P* = 0.20**	1	120	142	194	174	170	180	x	x	x	x	x	x				
	2	131	145	196	178	172	183	136	140	140	135	137	132				
	3	138	147	197	179	173	184	x	x	x	x	x	x				
	4	145	152	200	181	176	188	144	146	155	145	143	144				
	5	154	155	210	183	177	198	x	x	x	x	x	x				
	6	160	159	212	185	180	199	167	154	160	159	157	150				
	7	163	161	216	189	185	202	x	x	x	x	x	x				
	8	171	166	214	191	190	202	170	162	163	166	166	159				
	9	175	169	209	193	198	202	x	x	x	x	x	x				
	10	183	173	210	187	200	202	175	168	170	172	180	166				
	11	195	180	201	201	201	202	x	x	x	x	x	x				
	12	196	180	208	202	202	203	183	185	177	177	180	173				
	13	199	180	214	202	203	204	x	x	x	x	x	x				
	14	200	181	215	205	205	205	190	186	176	184	175	174				
	15	200	181	216	206	206	205	x	x	x	x	x	x				
	16	200	182	220	207	206	206	192	186	177	185	177	175				
	17	202	183	220	207	206	209	x	x	x	x	x	x				
	18	205	184	221	206	206	215	192	188	180	190	180	181				
	19	206	184	221	207	207	214	x	x	x	x	x	x				
	20	206	184	221	206	213	215	196	196	184	197	189	188				
	21	206	184	222	208	213	217	x	x	x	x	x	x				

	22	210	186	221	209	213	220	200	197	189	201	192	189
	23	211	187	221	210	213	220	x	x	x	x	x	x
	24	214	187	222	213	213	220	200	198	189	201	194	190
	25	214	187	222	215	213	220	x	x	x	x	x	x
	26	214	187	222	215	213	220	200	202	190	200	192	190
	27	214	190	222	217	215	220	x	x	x	x	x	x
	28	214	190	222	218	216	220	200	202	198	200	196	194
	29	214	193	222	219	219	220	x	x	x	x	x	x
	30	214	192	222	221	221	220	206	202	198	200	196	194
L. lagurus		C1	C2	C3	C4*	C5*	C6*	S1	S2	S3	S4	S5	S6
***P* = 0.17**	1	31	29	25	30	30	21	x	x	x	x	x	x
	2	32	31	25	31	33	23	22	24	23	22	26	27
	3	35	33	26	33	36	25	x	x	x	x	x	x
	4	36	36	27	34	39	26	26	29	28	26	30	29
	5	41	38	27	35	41	27	x	x	x	x	x	x
	6	38	39	29	34	40	33	32	30	34	33	35	33
	7	36	40	30	34	40	38	x	x	x	x	x	x
	8	39	42	33	35	40	40	30	32	34	35	37	37
	9	41	42	34	36	41	41	x	x	x	x	x	x
	10	42	42	34	36	41	42	33	35	35	39	36	39
	11	45	44	36	36	43	42	x	x	x	x	x	x
	12	45	44	36	38	43	42	39	38	36	40	35	39
	13	45	44	36	39	43	42	x	x	x	x	x	x
	14	45	44	36	40	43	42	40	38	37	40	35	43
	15	45	44	36	40	43	42	x	x	x	x	x	x
	16	45	45	36	42	43	42	44	38	40	39	35	42
	17	48	44	37	43	44	43	x	x	x	x	x	x
	18	49	43	38	44	46	43	43	39	40	40	37	42
	19	50	43	39	44	45	43	x	x	x	x	x	x
	20	50	45	38	44	45	43	42	38	40	40	38	43
	21	50	45	38	44	45	43	x	x	x	x	x	x
	22	50	44	38	44	45	43	46	39	40	40	38	42
	23	50	44	39	44	47	43	x	x	x	x	x	x
	24	50	44	39	44	46	44	46	39	40	41	37	44
	25	49	46	40	44	45	44	x	x	x	x	x	x
	26	49	46	40	44	47	44	47	44	41	43	40	49
	27	50	46	40	44	47	44	x	x	x	x	x	x
	28	50	46	40	44	46	44	46	44	42	45	41	50
	29	50	46	40	44	46	44	x	x	x	x	x	x
	30	50	46	40	44	46	44	47	45	43	45	40	50

CX, rodents inoculated with CDP, SX, rodents inoculated with SDP. *, animals inoculated with 10^5 parasites. P, significance of the difference between CDP and SDP groups in weight gains.

Table S5. *Leishmania* loads and distribution determined by qPCR in animals inoculated with *L. donovani*.

Rodent species	Tissues and organs	10^7 CDP							SDP						
**BALB/c mice** *P* = 0.27		**C1**	**C2**	**C3**	**C4**	**C5 ***	**C6**	**C7***	**S1**	**S2**	**S3**	**S4**	**S5**	**S6**	**S7**
	**B**	0	0	0	0	NA	0	NA	0	0	0	0	0	0	0
	**IE**	+	0	+	+	NA	+	NA	+	+	0	+	0	+	0
	**CE**	0	0	0	+	NA	0	NA	0	0	0	0	0	+	0
	**LN-IE**	0	0	+	+	NA	+	NA	+	0	0	0	0	+	0
	**LN-CE**	0	0	0	+	NA	0	NA	0	0	0	0	0	+	0
	**FP**	0	0	0	+	NA	0	NA	0	0	0	0	0	0	0
	**HP**	0	0	0	+	NA	0	NA	0	0	0	0	0	+	0
	**T**	0	0	0	0	NA	0	NA	0	0	0	0	0	0	0
	**L**	0	0	0	++	NA	+	NA	0	0	0	+	0	++	0
	**S**	0	0	+	+	NA	+	NA	+	0	0	+	0	+	0
C griseus *P* = 0.51		**C1**	**C2**	**C3**	**C4**	**C5**	**C6**	**C7**	**S1**	**S2**	**S3**	**S4**	**S5**	**S6**	**S7**
	**B**	0	0	0	0	0	0	0	0	0	0	0	0	0	0
	**IE**	+	0	+	+	0	+++	+	+	+	+	++	+++	++	0
	**CE**	0	0	0	0	0	+	+	0	0	0	0	+++	0	0
	**LN-IE**	0	0	+	+	0	++	0	+	+	+	0	++	++	0
	**LN-CE**	0	0	0	0	0	0	0	0	0	+	0	0	0	0
	**FP**	0	0	+	0	0	++	0	0	+	0	+	++	0	0
	**HP**	0	0	0	0	0	++	0	0	0	+	+	0	0	0
	**T**	0	0	0	0	0	0	0	0	0	+	0	0	0	0
	**L**	++	++	0	0	0	++	0	0	++	0	++	++	0	0
	**S**	+	+	++	0	0	++	+	+	+	+	++	+++	0	0

Rodent species	Tissues and organs	10^7 CDP			10^5 CDP			SDP					
M. auratus *P* = 1.00		**C1**	**C2**	**C3**	**C4**	**C5**	**C6**	**S1**	**S2**	**S3**	**S4**	**S5**	**S6**
	**B**	0	0	0	0	0	0	0	0	0	0	0	0
	**IE**	+	0	+	+	0	0	0	+	+	+	+	+
	**CE**	0	0	0	0	0	0	0	0	0	0	0	0
	**LN-IE**	0	0	+	0	0	0	0	0	0	0	0	0
	**LN-CE**	0	0	0	0	0	0	0	0	0	0	0	0
	**FP**	0	0	0	0	0	0	0	0	0	0	0	0
	**HP**	0	0	0	0	0	0	0	0	0	0	0	0
	**T**	0	0	0	0	0	0	0	0	0	0	0	0
	**L**	0	0	0	0	0	0	0	0	0	0	0	0
	**S**	0	0	0	0	0	0	0	0	0	0	0	0
L. lagurus *P* = 0.21		**C1**	**C2**	**C3**	**C4**	**C5**	**C6**	**S1**	**S2**	**S3**	**S4**	**S5**	**S6**
	**B**	0	0	0	0	0	0	0	0	0	0	0	0
	**IE**	+	+++	+++	+	0	0	+++	+++	++	+++	+++	+++
	**CE**	+	++	+	0	0	0	+	+	0	++	0	++
	**LN-IE**	+	++	++	0	0	0	+	++	0	++	++	++
	**LN-CE**	0	+	+	0	0	0	+	0	0	+	++	++
	**FP**	+	++	++	+	0	0	++	+	0	+++	++	++
	**HP**	+	++	+++	0	0	0	++	0	0	++	++	0
	**T**	0	+	+	+	0	0	+	+	0	+	+	0
	**L**	+	+++	++	+	0	0	+++	+++	0	+++	+++	+++
	**S**	++	+++	+++	+	0	0	+++	++	0	+++	+++	+++

10^7 CDP - Rodents inoculated with 10^7 culture-derived parasites, 10^5 CDP - Rodents inoculated with 10^5 culture-derived parasites, SDP - rodents inoculated with sand fly -derived parasites. *, animals dying before the end of the experiment. NA, not analyzed, IE, inoculated ear; CE, contralateral ear; LN-IE, draining lymph nodes of the inoculated ear; LN-CE, draining lymph nodes of the contralateral ear; FP, forepaws; HP, hindpaws; T, tail; L, liver; S, spleen; B, blood; +, $< 10^3$ parasites; ++, 10^3 -10^4 parasites; +++, $> 10^4$ parasites. P, significance of the difference between CDP and 10^7 SDP groups.
